# Biodegradation and Thermomechanical Behavior of 3D-Printed PLA Scaffolds Under Static and Stirring Biomimetic Conditions

**DOI:** 10.3390/biomimetics9120743

**Published:** 2024-12-05

**Authors:** Diana V. Portan, Georgios Bampounis, Athanasia Koliadima, Anastasios C. Patsidis, Lykourgos C. Kontaxis, George C. Papanicolaou

**Affiliations:** 1 Department of Mechanical Engineering and Aeronautics, University of Patras, 26504 Patras, Greece; geobampou@gmail.com (G.B.); gpapan@upatras.gr (G.C.P.); 2 Physical Chemistry Laboratory, Department of Chemistry, University of Patras, 26504 Patras, Greece; akoliadima@upatras.gr; 3 Department of Materials Science, University of Patras, 26500 Patras, Greece

**Keywords:** 3D-printed PLA scaffolds, biodegradation, dynamic mechanical analysis (DMA), differential scanning calorimetry (DSC)

## Abstract

3D-printed biomedical polylactic acid (PLA) scaffolds were developed, and their biodegradation, as well as their thermomechanical behavior, were studied in a relevant in vitro environment. The scaffold’s biodegradability profile has been monitored after immersion in a cell culture medium that contains components of blood and body fluids. Two types of biodegradation experiments were performed—a standard static one and an adapted stirring one, mimicking the body fluids’ flow, respectively—to achieve a comparative investigation. The biodegradation experiment’s duration was one month. The measurements were performed between days 1 and 28. The scaffold microstructure was analyzed with scanning electron microscopy (SEM). The weight loss of the scaffolds has been monitored. Differential scanning calorimetry (DSC) has been used to evaluate the glass transition temperature (T_g_) of the scaffolds and to draw useful conclusions about their thermal behavior. Finally, dynamic mechanical analysis (DMA) was applied to investigate the viscoelastic behavior of the samples. The SEM analysis demonstrated that the samples in a static experiment are more damaged, while those in the stirring experiment are more brittle. The maximum T_g_ value of the material measured by DSC is around 65 °C. This value is reached after 5 days of immersion in static conditions and after 14 days of immersion after stirring, indicating that some processes take place faster in the static experiment. The variation of the T_g_ vs. immersion time, as derived from DSC vs. DMA measurements, gives similar results for both static and fluid absorption conditions, demonstrating the reproducibility of the results.

## 1. Introduction

Three-dimensional scaffolds have been intensively used in vitro for the last decades, being employed as innovative and convenient platforms for the growth and long-term maintenance of human cell cultures [1,2]. The purpose of the in vitro research associated with scaffolds is, among others, to clarify the crucial aspects related to cell populations’ basic biology; furthermore, creating a third dimension for cell culture is more relevant, but the analysis of this complex system requires a multidisciplinary approach [3]. Although, in scientific literature scaffolds are promoted in relation to biomedical applications, a lot of in vitro data related to their response to a physiologic-like environment is missing. The ‘bench to bedside’ shifting can be succeeded only after performing complex in vitro investigations of the scaffold’s response to different micro-environmental stimuli. The prediction of scaffold behavior upon implantation is crucial, since its instant breakage or failure in an undesired mode may cause great damage to the tissue that has naturally grown around and within it. A possible failure will depend on many parameters, such as material type, architecture, and dimensions, as well as on the loading at situs of implantation under mechanical fatigue. In order to avoid chaotic in vivo events, appropriately configured materials should be fabricated to modulate the different stages of the healing response by inducing a shift from a process of inflammation and scar tissue formation to one of constructive remodeling and functional tissue restoration [4].

Understanding the biodegradation profile of a scaffold and the decline of its properties is extremely important when it comes to successful application in implantology. The mechanical characterization of the scaffolds upon long-term immersion in simulated body fluids can offer information on its feedback when exposed to micro-environments that closely mimic the physiological one. The body micro-environment involves: (1) humidity, due to the existence of the body fluids, which influence the radical processes taking place in a biomaterial, changing pathways and reaction kinetics [5]; (2) a physiological condition with comparable ranges of load or pressure, viscosity, and sliding speed, which produces friction and complex tribological phenomena, depending on the tissue location [6]; (3) low-intensity electricity generated by the human body through the biological electric field and current electrical potentials, ranging between 10 and 60 mV at various locations [7]; (4) vibrations produced by cyclic loading of the tissues that have elastic properties [8]; and (5) complex mechanical loading, especially in the case of the musculoskeletal system. All these factors degrade the biomaterial, while the body temperature accelerates the damaging processes by inducing an ageing effect [9], which is extremely pronounced in the case of thermoplastics [10]. Moreover, the porous architecture of scaffolds ends up in robustness loss, followed by faster decomposition. The yielding of the scaffold’s layer lines due to the above-mentioned ageing parameters determines the change in geometry and, in vivo, it may delocalize tissue at the situs of implantation, enabling several unpredicted situations.

Biodegradability is a property of high interest associated with biomedical scaffolds because it directly affects the degradation of the mechanical properties of a biomaterial. The degradation of scaffolds has been investigated previously, especially in the case of metallic materials, to evaluate their corrosive behavior and the loss of mechanical performance. For instance, it has been shown that porous scaffolds made of metallic alloys corrode too rapidly in artificial media, because of the high chemical activity of their elements and large surface. The rapid degradation of magnesium and its alloys gives rise to the loss of mechanical integrity, the generation of harmful hydrogen cavities, and local alkalization around the implants. Some other alloys have been proposed with improved corrosion resistance [11], but the situation has not improved considerably, thus limiting the application of these types of materials. The biodegradation of polymeric scaffolds is affected by different mechanisms compared to metallic scaffolds. Few studies are dedicated to thermoplastic scaffold biodegradation [12,13]. It is considered that the effect of the fluid flow [14] in the body is decisive for thermoplastic in vivo performance. Furthermore, body temperature is the main influencing factor involved in thermoplastic biodegradation.

Polylactic acid (PLA) is the most common biomedical thermoplastic; in addition, it is FDA (Food and Drug Administration)-approved, which makes it suitable for application in contact with human tissues. However, similar to most thermoplastic polymers, it can be mechanically weak when exposed to complex loading and humidity [15]. Special attention has been paid to the structural relaxation that can occur in blends with polylactic acid at body temperature, which may change the physical properties of the material during its application as a biomaterial [16]. Two separate mechanisms have been identified as being responsible for PLA deterioration, as follows: bulk erosion, which is characterized by uniform degradation across the polymer’s surface, and surface erosion, which is concentrated at the interface between the polymer and water. The two processes that lead to PLA degradation are water absorption and ester bond hydrolysis. The phases of (i) water diffusion into the polymer, (ii) polymer–fluid chemical interaction, and (iii) the leaching of the resulting low-molecular-weight species are also included in the kinetics of the different processes. Furthermore, there is debate in the literature on the effect of PLA’s initial degree of crystallinity on its rate of degradation. Despite the high interest in PLA and other thermoplastics used in scaffold manufacturing, the investigation of their thermomechanical properties when performing in a relevant environment has been neglected, especially for long-term exposure. Nevertheless, one disadvantage of 3D-printed polymeric materials is that moisture could seep between the layers and holes of the final product. More precisely, during the printing process, steam or bubbles are created by the filament’s moisture, which results in uneven extrusion and layer adhesion. These flaws may, therefore, impair the printed object’s overall quality and aesthetics, influencing both its final appearance and structural integrity [17]. Understanding the deterioration brought on by fluids is essential to expanding the range of uses for common 3D-printed materials.

The investigation of phase transition issues in the biomedical field is required, because the study of thermodynamic parameters, such as the glass transition temperature (T_g_), melting temperature (T_m_), crystallization temperature (T_c_), enthalpy (ΔH), and heat capacity (C_p_), may provide important information that can be used in the development of new products. The most employed techniques for characterizing phase transitions are thermogravimetric analysis (TGA), dynamic mechanical analysis (DMA), thermomechanical analysis (TMA), and differential scanning calorimetry (DSC). Among these techniques, DSC is preferred, because it allows for the detection of transitions in a wide range of temperatures (−90 to 550 °C) and eases the quantitative and qualitative analysis of the transitions [18]. Differential scanning calorimetry is a useful tool for studying the thermophysical properties of biomaterials in solutions. It covers specific methods used to study thermal processes, including crystallization, formation, glass transition, recrystallization, melting, molecular relaxation, and phase separation [19]. Furthermore, the DMA technique is a very suitable tool to investigate the viscoelastic properties of polymers in a wide range of temperatures and frequencies and has been used on polymeric biomaterials [20]. Dynamic mechanical methods enable accurate and rapid quantification of the viscoelastic properties of pharmaceutical and biomedical systems by the analysis of the loss and storage moduli in correlation to the micro-environment [21].

The aim of the present investigation is to monitor the behavior of 3D-printed PLA scaffolds with respect to their biodegradation profile and their thermomechanical response when subjected to a relevant environment. A comparison of the biodegradation mechanism of scaffolds immersed in immobilized body fluid (static conditions)—which do not replicate the body micro-environment upon implantation—and the corresponding biodegradation behavior when immersed in a rotating fluid environment created using a stirrer (stirring conditions)—which partially reproduced the body micro-environment parameters—was made possible by the design of the experimental setup used in this study. The tests were performed at body temperature. The regularly used simulated body fluid (SBF), composed of water and salts, has been replaced in this study by a cell culture medium that is richer in biochemical elements and closely reproduces the blood components, as well as fluids in the human body, for a better assembly of real body conditions. The gain/loss of weight of the scaffolds has been monitored, followed by an investigation of their changes in the thermomechanical properties using DSC and DMA. The correlation of the results elucidates the significance of establishing appropriate experimental conditions for the in vitro investigation of biomaterials, which closely mimic the human body environment, highlighting the impact of these conditions on the behavioral variations observed when compared with non-relevant testing methodologies.

## 2. Materials and Methods

### 2.1. Materials

Natural PLA with a diameter of 1.75 mm from Innofil3D (Emmen, The Netherlands) has been used for the manufacturing of compact and porous PLA specimens. A total of 21 samples were used for the experiment of static degradation and another 21 samples were used for the dynamic degradation experiment (stirring). The cell culture medium has been freshly prepared to immerse the samples for several days (0, 1, 3, 7, 10, 14, and 21). The cell culture medium consists of the following: Alpha-Minimal Essential Medium (a-LEL, BiochromKG, seromed, Berlin, Germany), with 10% fetal bovine serum (FBS, Biochrom), supplemented with 2.5 μg/mL Amphotericin B (Biochrom), 50 μg/mL Gentamycin (Biochrom), 1.5 mM B-glycerophosphate (Sigma, Athens, Greece), 50 μg/mL Ascorbic Acid (Sigma), and 50 μg/mL Penicillin-Streptomycin [22,23]. The composition of the cell culture medium is normally employed to maintain stem and bone cells (osteoblasts) in vitro. This medium offers the necessary elements that enable cells’ survival and proliferation.

### 2.2. Methods

**Figure 1:**
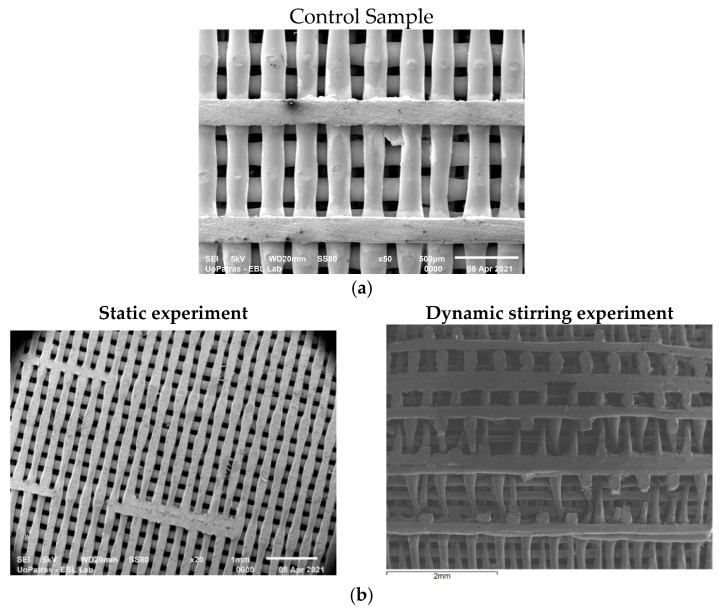

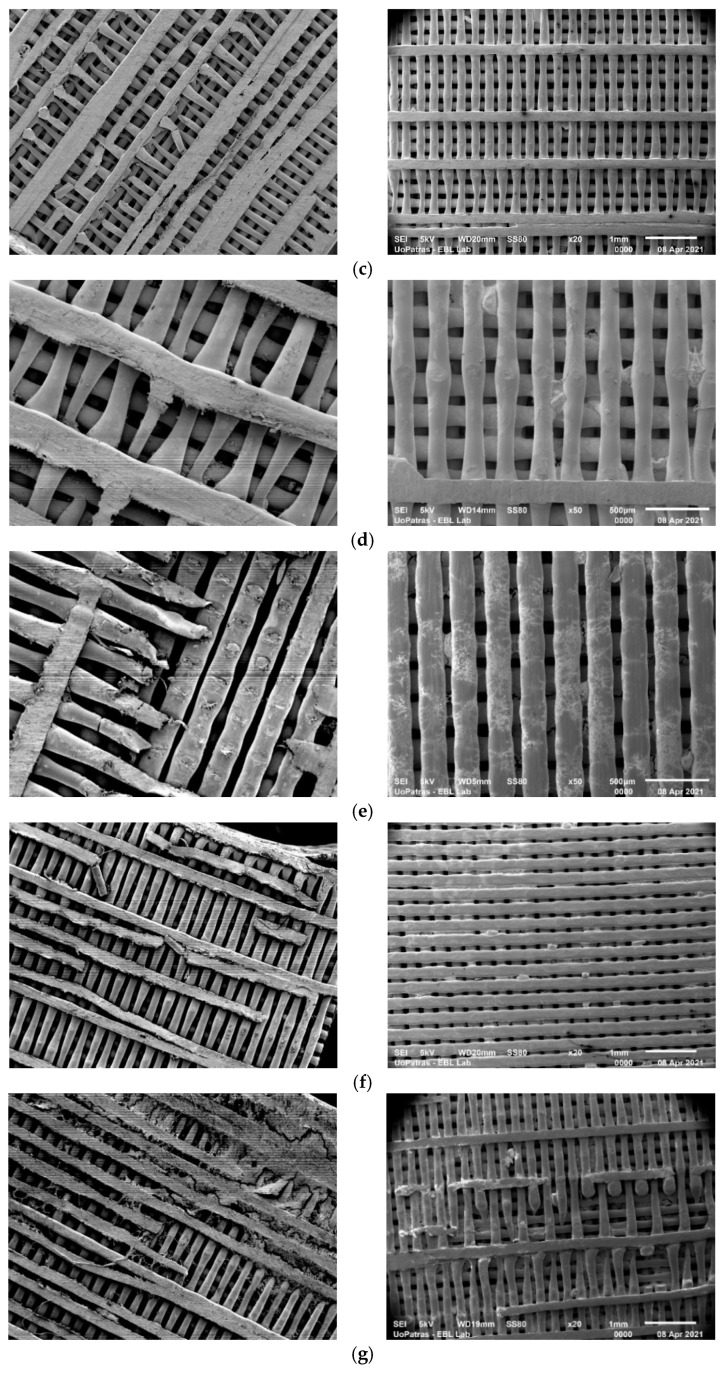

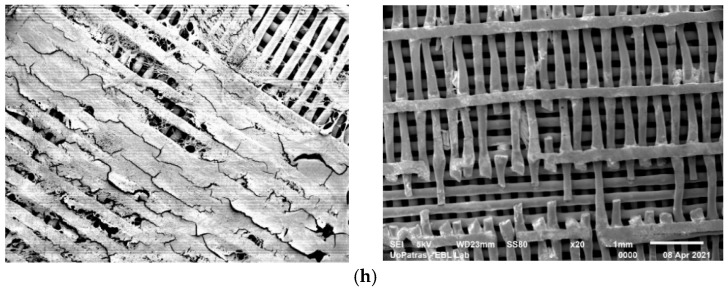
SEM analysis of scaffolds subjected to static biodegradation (images on the left side) and those subjected to dynamic stirring biodegradation (images on the right side), at different magnifications and stages of their immersions: (**a**) 50×, 0 days of immersion; (**b**) 20×, 1 day of immersion; (**c**) 20×, 3 days of immersion; (**d**) 50×, 7 days of immersion; (**e**) 50×, 10 days of immersion; (**f**) 20×, 14 days of immersion; (**g**) 20×, 21 days of immersion; (**h**) 20×, 28 days of immersion.

A 3DISON AEP desktop 3D printer (ROKIT, Seoul, Republic of Korea) based on fused filament fabrication (FFF) has been used to manufacture the specimens. The key printing parameters were selected, as described in [24], as follows: 190 °C nozzle temperature, 40 °C chamber temperature, 30 °C bed temperature, layer height of 0.12 mm, and a low printing speed (20 mm/s). The scaffolds were designed with square-shaped pores and a side length of 100 μm; the layers were perpendicular to each other (0°/90°), and their dimensions were 10 × 10 × 5 mm^3^. According to the 3D printing software calculations, the samples had a porosity volume of 39.16% and, based on our previous findings, their stiffness was around 177 MPa. The pores’ structure of the scaffold can be seen in Figure 1a. A JSM-6610 Series scanning electron microscope (SEM) model has been used to analyze the samples at different magnifications. Parts of the immersed samples were removed and used for the DSC and the DMA tests.

The description of the experimental setups for degradation is given below, as follows:
  (1) Setup for the static degradation experiment: A total of 60 mL SBF was added to a Berzelius beaker. Subsequently, 21 scaffolds were immersed in the fluid. The beaker was then placed in an incubator and maintained at a temperature of 37 °C, standard for the human body. Aluminum foil was used to prevent the liquid from evaporating. The aluminium foil was perforated with tiny holes to promote oxygenation and eliminate anaerobic contamination.
  (2) Setup for the dynamic degradation experiment: The dynamic experiment involved active stirring. As in the case of the static experiment, 60 mL SBF was placed in a Berzelius beaker. A total of 21 samples were immersed in the solution together with two rotating magnets. The beaker was then placed on a magneto-thermo stirrer. The temperature of the solution was maintained constant at 37 °C and regularly checked with a thermometer. Aluminum foil was used to prevent the liquid from evaporating. To allow oxygenation, small holes were made in the aluminum foil. The liquid with the scaffolds was in constant motion at 500 RPM.

#### 2.2.1. Weight Loss Evaluation

For the evaluation of weight loss, 3 samples were extracted from the fluid for each testing day (1, 3, 7, 10, 14, and 21). The samples were dried for 15 min with an air drier, which was placed at a steady 50 cm distance from the sample. The percentage of weight loss after immersion has been calculated.

#### 2.2.2. DSC Measurements

Differential scanning calorimetry was employed to investigate the thermal response of the scaffolds. Approximately 3 mg of each specimen was put into aluminum crucibles, which were then hermetically sealed and tested in a Q200 (TA Instruments, New Castle, DE, USA) apparatus under a nitrogen atmosphere at a flow rate of 50 mL/min, and from 25 to 100 °C at a heating rate of 10 °C/min. The T_g_ of the samples was assessed by analyzing the DSC curves.

#### 2.2.3. DMA Measurements

The dynamic mechanical behavior was assessed by conducting DMA experiments utilizing a TA Q800 (TA Instruments, New Castle, DE, USA) apparatus, according to ASTM D5023 guidelines. The dimensions of the specimens were 50 × 12.8 × 3.2 mm^3^ while the three-point bending mode was utilized. In the first heating cycle, the following experimental parameters were used: 25–120 °C temperature range, 1 Hz frequency, 10 μm amplitude, and 2 °C min^−1^ heating speed.

#### 2.2.4. Morphological Analysis

A scanning electron microscope JSM-6610 (JEOL, Toky, Japan) was used to analyze the surface morphology and architecture of the 3D-printed PLA scaffolds. The scaffolds’ microphotographs were taken under a vacuum with a voltage of 2 kV after being coated with gold using a sputter coater (Bal-Tec SCD005, Balzers, Liechtenstein).

## 3. Results

### 3.1. SEM Analysis

Micrographs of the samples at different stages of biodegradation are shown in the SEM photos in Figure 1. The images in the left column are of samples exposed to the static experiment, while, in the right column, images of samples that were subjected to stirring are presented. In Figure 1a, a control pristine sample is presented (0 days of immersion). It can be observed that the stirring experiment resulted in the visible rupture of small parts of the layer lines in the scaffolds after one day of immersion and, apparently, the dynamic experiment impacts the scaffold structure more than the static one. However, after several days of immersion, the samples in the static experiment were visibly more damaged than the stirring ones.

### 3.2. Weight Loss

**Figure 2:**
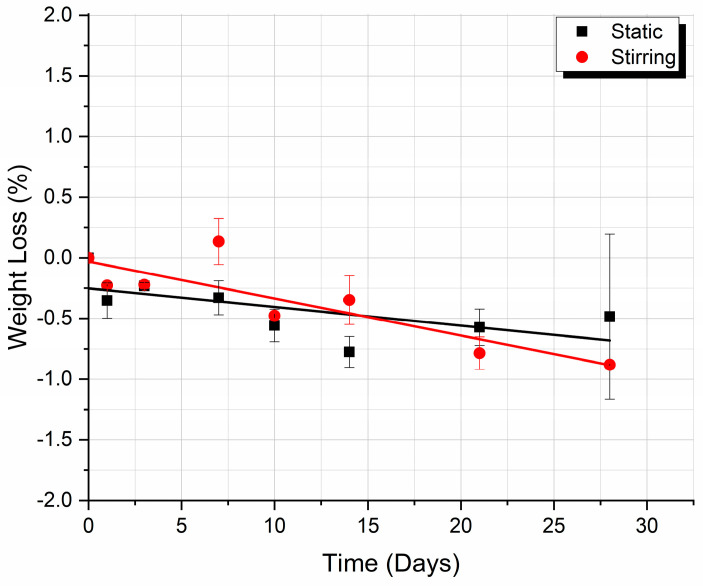
Diagram of the weight loss percentages in samples after different immersion times and for the two types of experiments (static and dynamic stirring, respectively).

As shown in the diagram in Figure 2, in the case of both experiments—static and stirring—samples lose weight after immersion in the cell culture medium, entering a degradation process after one day of immersion. More precisely, the amount of absorbed fluid does not contribute enough to their mass increase to surpass their initial weight. After approximately two weeks of immersion, the weight of the static and dynamic immersed samples coincide. More discussion on the processes that influence weight loss variation during biodegradation is given in Section 4.2.

### 3.3. Differential Scanning Calorimetry (DSC)

**Figure 3:**
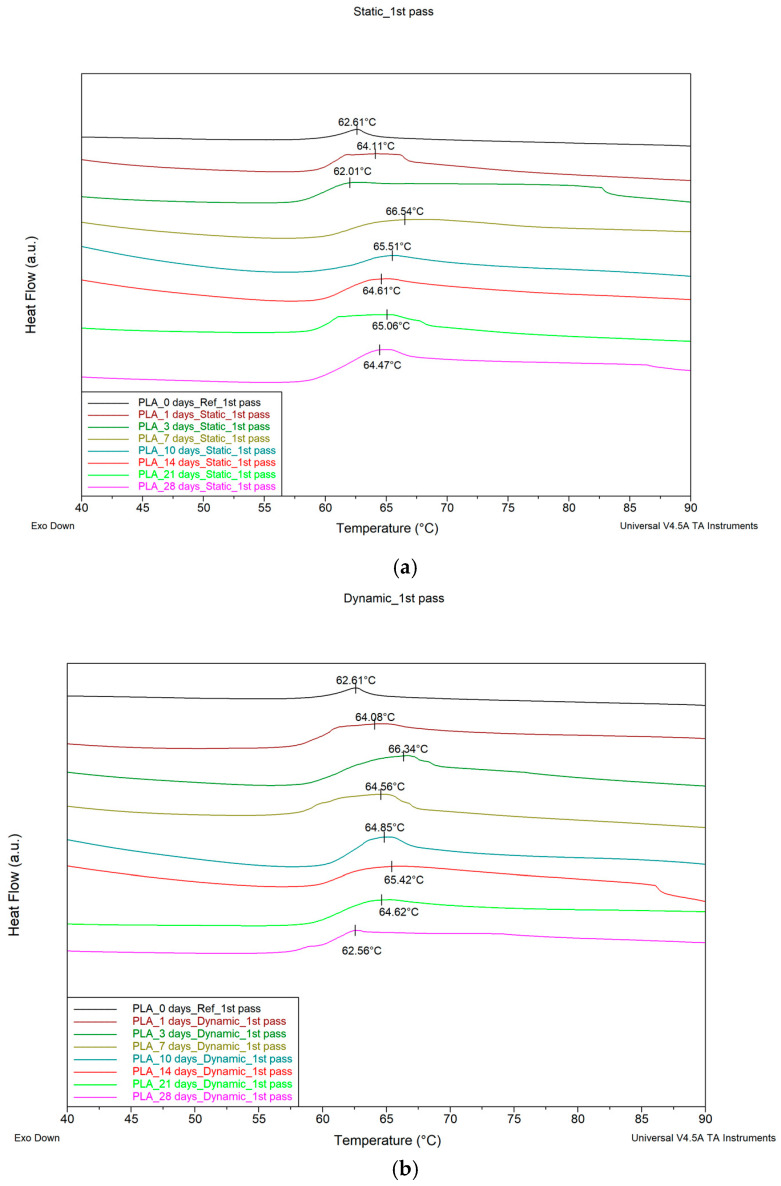

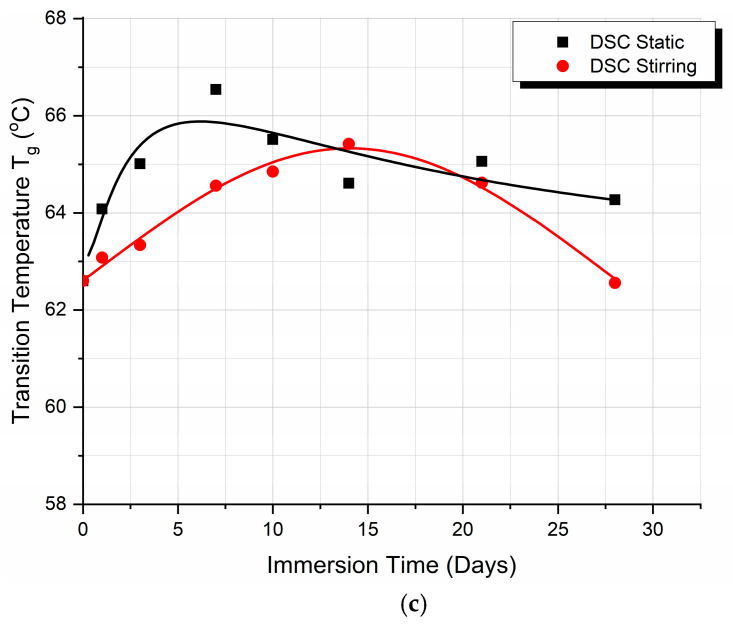
DSC thermograms for PLA specimens for different immersion durations under (**a**) static conditions, (**b**) stirring conditions, and (**c**) glass transition temperature (T_g_) value variation with immersion time when under static and stirring conditions.

Figure 3a,b show DSC thermograms for the PLA samples that have absorbed fluid at different times of immersion when under static fluid absorption (static) and stirring absorption conditions (dynamic), respectively. Since we would like to mimic the real application conditions (i.e., SBF absorption effect) of the material, the 1st pass of the DSC runs was used. The variation of the T_g_ value with time of immersion in both cases is shown in Figure 3c.

The T_g_ of the material for static immersion initially increases with immersion time, attaining after five days of immersion a maximum value of approximately 65.8 °C, and then decreases at a relatively slow rate. In contrast, in the case of stirring immersion, an initial increase in T_g_ is observed up to about 65 °C, followed by a faster decrease rate as the time of immersion increases. The maximum T_g_ value under stirring immersion conditions was attained after 14 days of immersion.

### 3.4. Dynamic Mechanical Analysis (DMA)

**Figure 4:**
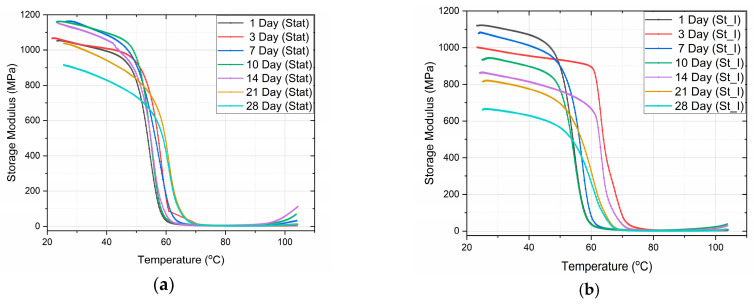

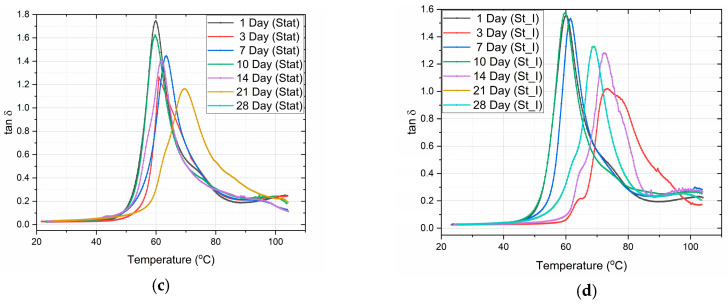
Storage modulus vs. temperature for (**a**) static and (**b**) stirring conditions. Also, tanδ vs. temperature for (**c**) static and (**d**) stirring conditions.

In Figure 4, the variation of both the storage modulus and the tanδ of PLA specimens vs. the temperature for static and stirring conditions is shown, as measured by DMA. The glass transition temperature was determined as the temperature corresponding to the peak of the tanδ curves.

**Figure 5:**
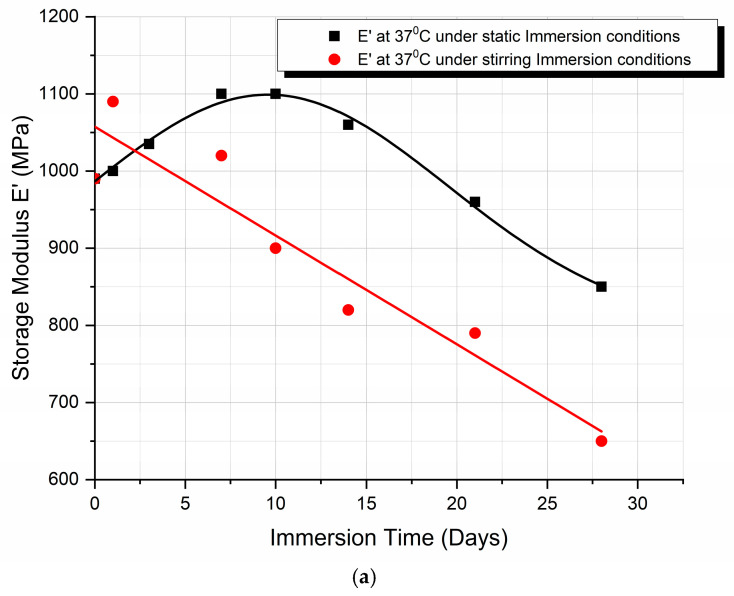

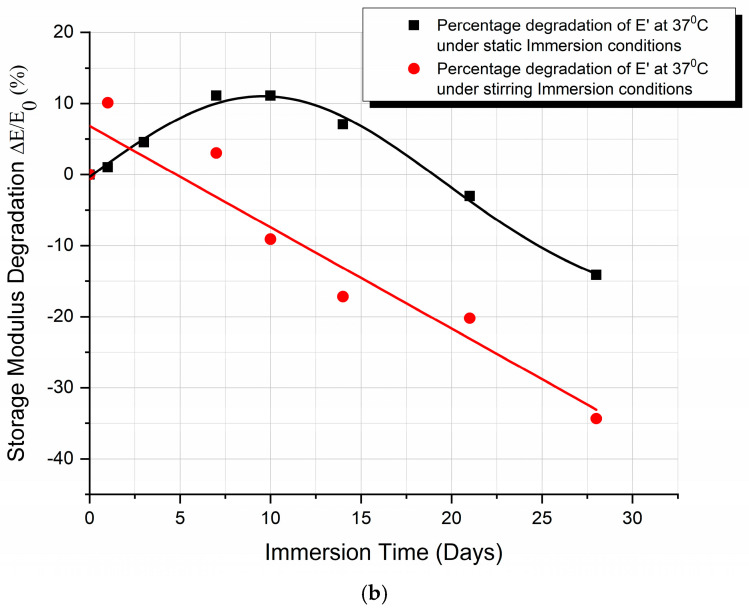
Comparison of (**a**) the storage modulus E′ variation and (**b**) the percentage storage modulus degradation, with immersion time, under static and stirring absorption conditions at 37 °C.

A comparison between the storage modulus E′ variation and the percentage storage modulus degradation, with immersion time, when under static and stirring absorption conditions at 37 °C, is shown in Figure 5a and Figure 5b, respectively.

**Figure 6:**
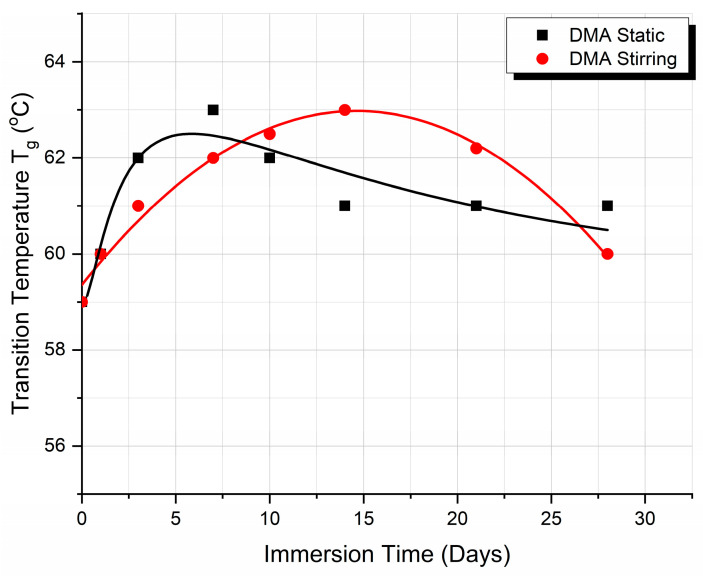
DMA glass transition temperature (T_g_) variation with immersion time when under static and stirring conditions.

Finally, the variation of the T_g_ vs. the immersion time, as derived from the DMA measurements, for both static and fluid absorption conditions, is shown in Figure 6. It can be observed that the two curves are similar in shape to those presented in Figure 3c, derived from the DSC experiments, demonstrating the reproducibility of the results.

### 3.5. DMA and DSC Comparison

**Figure 7:**
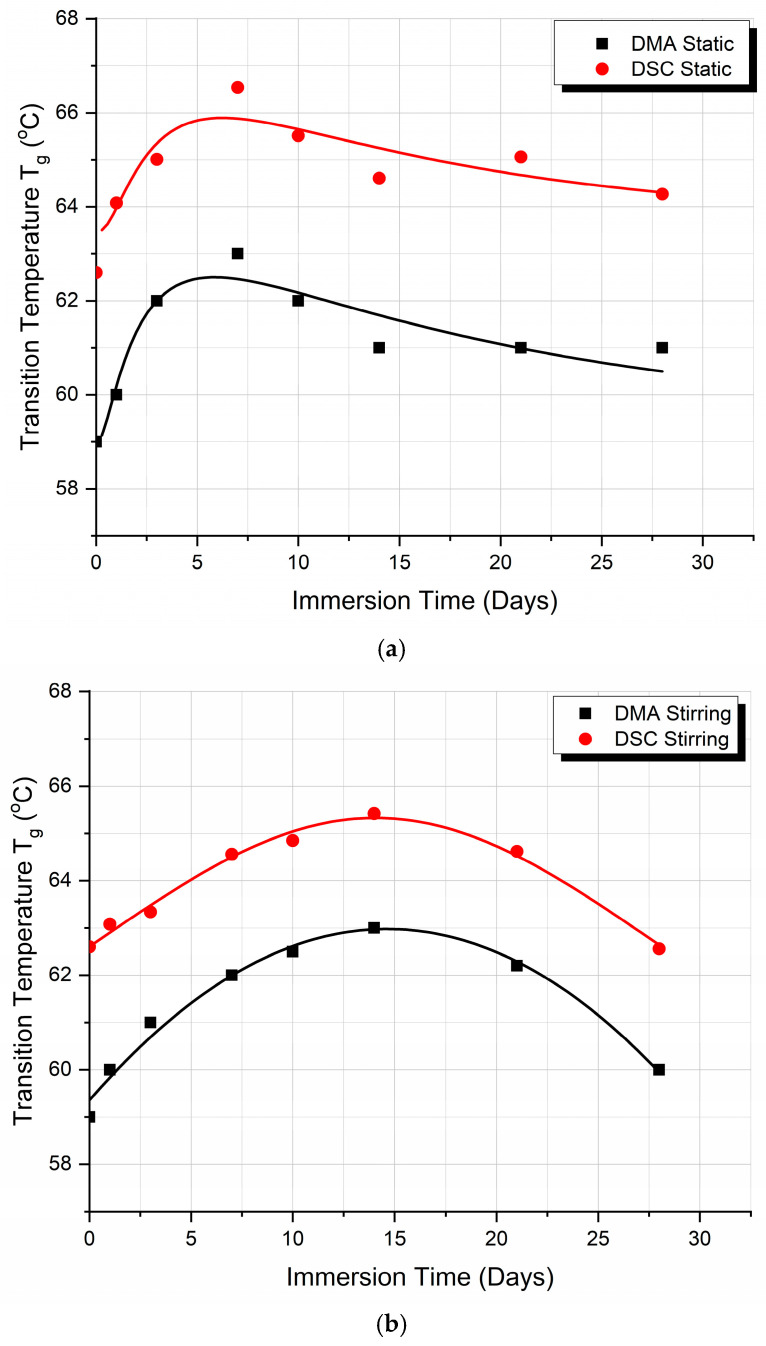
Comparison between T_g_ values as derived from DMA and DSC experiments: (**a**) under static absorption conditions and (**b**) under stirring absorption conditions.

Figure 7 compares the T_g_ values of the PLA specimens tested by DSC and DMA methods for both static and stirring conditions. In Figure 7a, a comparison between DSC and DMA methods for the static results is presented, while, in Figure 7b the same comparison is made for the stirring case.

## 4. Discussion

### 4.1. Structural Changes Observed in SEM Micrographs

As seen in Figure 1, layer line cracks are observable (Figure 1, dynamic stirring) in the case of the samples subjected to dynamic stirring from the first day of immersion. These cracks may also be seen in the static sample after 3 days of immersion (Figure 1c, static stirring). However, the cracks observed on the first day of dynamic stirring are much smaller and fewer in number than those formed in the static experiment after 10 days of immersion. These small cracks created during the first day of immersion may be associated with imperfections acquired during the fabrication process, since the time of immersion is too short to attribute the specific events to the absorption and degradation processes. For all the other immersion days, it was observed that the samples are much more affected by fluid absorption in the case of the static experiment, denoting that some complex phenomena happen at the molecular level when motion is applied. More precisely, competitive processes take place during immersion, which affect the weight of the scaffolds, as follows: (a) the degradation of the PLA polymer and (b) the absorption of the biological fluid by the polymer. Furthermore, in both experiments, a leaching phenomenon takes place, but with different consequences in each one. In the stirring immersion experiment, the released polymer molecules escape into the surrounding liquid, while, in the static experiment, they remain attached onto the polymer surface. Thus, weight loss is greater in the stirring experiment. Specimen rupture becomes evident after 10 days of immersion under static conditions. On day 21 of immersion, the sample under static conditions presents both layer line rupture and layer line dilution. The samples immersed under dynamic conditions maintain the initial shape of their lines after 28 days of immersion, and the number of dislocations is visibly lower than that observed in the case of the samples exposed to static degradation. Also, plasticization is evident in the scaffolds maintained under static conditions.

The significant difference in the immersion effect on the samples in static vs. dynamic modes is enabled by the position of the samples in the Berzelius beaker during the experiments and the mixing of the nutrients in the fluid, which are key elements that guide the entire process. The samples in the static experiment stay at the bottom of the beaker, and amino acids, salts, as well as other elements in the fluid, gradually deposit on the layer line surface, determining a steady corrosion rate. On the other hand, the stirred samples are moving constantly, and, due to the rotation forces, the deposition of different nutrients on their layer lines is delayed. Also, they are maintained at mid-surface levels in the beaker compared to the static samples, which are at the bottom.

Another important factor affecting the degradation of PLA scaffolds, due to water absorption, is their porosity and pore size. Odelius, et al. [25] postulated that even though porous scaffolds and solid films are thin, the thickness of the solid material between the pores will influence the deterioration rate due to the varying pore sizes. Because thinner pore walls are the result of smaller pores, the rate of degradation will also be lower. There will be more pores and pore walls with smaller pore diameters, though, and the degradation products that are produced will have a longer migration path through the scaffold to the surrounding media. In conclusion, the smaller the pore size, the slower the hydrolysis.

### 4.2. Weight Loss Analysis

The weight loss of the samples in an aqueous medium reflects the release of soluble small molecules formed during the polymer hydrolysis reaction. The weight loss measurement is the first step in the investigation of biomaterial degradation. The first important parameter influencing the weight loss of the biomedical scaffolds immersed in the physiological fluid is modulated degradation. It is expected that, under dynamic stirring conditions, thermoplastics will achieve similar degradation profiles within a shorter period, when compared with those under static conditions. However, these conditions only partially mimic the actual situation, and subsequent analyses of derived mechanisms must be treated with caution and should always be supported by actual long-term degradation data obtained under physiological conditions. Previous studies have revealed that polycaprolactone (PCL) and PCL composite scaffolds degrade very differently under relevant degradation conditions, while still undergoing hydrolysis. The molecular weight and mass loss results differ due to the different degradation pathways followed (surface degradation pathway for accelerated conditions and bulk degradation pathway for simulated physiological conditions). Crystallinity studies have revealed similar patterns of recrystallization dynamics, and mechanical data have indicated that the scaffolds retained their functional stability, in both instances, over the course of degradation. Ultimately, polymer degradation was shown to be chiefly governed by molecular weight, crystalline susceptibility to hydrolysis, and device architecture considerations, while maintaining its thermodynamic equilibrium [26].

A second crucial parameter that influences weight variation is the type of physiological fluid that is used in the in vitro study. Generally, simulated body fluids like Hank’s solution or phosphate buffer saline are used. SBF is an acellular, protein-free, supersaturated calcium phosphate solution with an ionic composition nearly equal to that of human blood plasma and generally buffered at physiological conditions (pH 7.4 and 36.5 °C) [27]. Due to the lack of proteins and other elements in the SBF, the immersion experiments of biomaterials in these types of solutions partially reflect the reality of what happens when biomaterial meets body fluids. Compared to SBF, the cell culture medium used to maintain human or animal cells is much more relevant. It contains several biochemical elements. A typical culture medium is composed of a complement of amino acids, vitamins, inorganic salts, glucose, and serum as a source of growth factors, hormones, and attachment factors. In addition to nutrients, the medium also helps to maintain pH and osmolality. Consequently, it affects differently, through more complex biochemical processes, the degradation of the tested biomaterial. The significant change in biomaterial behavior, depending on the type of SBF, has been proved by Domingos et al. [28]. Weight loss percentages, as a function of time, of PCL scaffolds incubated in two types of artificial fluids (SBF and PBS, respectively) were investigated. The degradation in SBF determined a weight loss of about 1% due to the erosion of PCL, while the salt deposit began to influence the weight loss measurements for degradation times of more than 2 months. A PCL scaffold studied in PBS determined a much lower weight loss from 0.2 to 0.4%, which was kept constant up to the end of the experiment. For the present investigation, the selected artificial fluid was the cell culture medium, due to its richness in biochemical components; in addition, the purpose was to mimic the real body micro-environment in a better way.

The two key events that play an important role in the response of the scaffolds to the micro-environment are as follows: (1) material degradation and (2) fluid absorption. The static experiment is firstly governed by fluid and chemical intense absorption/adsorption, which leads to material loss and pronounced degradation of the macromolecules, however, at the same time, maintains the samples’ weight higher because an amount of fluid is entrapped into the material. The dynamic stirring experiment determined less fluid adsorption/absorption in the samples and a decrease in weight because the layer line rupture is more evident since the mass lost is not complemented by the water uptake phenomenon. On day 15, the weight is similar for the samples in both the static and the dynamic stirring experiments. Also, it should be noted that the transition temperatures of the samples coincide on day 15, as measured by DSC (Section 4.3), which needs further interpretation, as provided below.

### 4.3. Differential Scanning Calorimetry (DSC) Discussion

DSC is a thermodynamic technique that allows for the direct evaluation of heat energy uptake that takes place in a sample under controlled temperature increase or decrease. One common use for calorimetry is to track phase transition changes. The study of biological processes, which are defined as a single molecular transition of a molecule from one conformation to another, is a common application for DSC. The materials’ melting points, or thermal transition temperatures, are evaluated in their solid, liquid, or mixed phases. A basic DSC experiment involves simultaneously introducing energy into a reference cell (which is empty) and a sample cell (which includes a small sample of the material of interest). Both cells’ temperatures are gradually raised at the same rate. The amount of extra heat absorbed or emitted by the sample (during an endothermic or exothermic operation, respectively) would be the difference in the input energy needed to bring the sample’s temperature up to that of the reference. The idea is that excess heat enters the picture because it takes more energy to get the sample to the same temperature as the reference when the material of interest is present.

According to the theory, three competitive phenomena occur in thermoplastics, which affect their glass transition temperature T_g_, as follows:
  - The first phenomenon is that the absorption of moisture by the polymer enables plasticization. The macromolecules of the polymer chains acquire greater mobility. Therefore, the material needs less energy to change from the rigid state (glassy state) to the elastomeric state (rubbery state). Consequently, the T_g_ value decreases.
  - The second phenomenon concerns the change in the density of the polymer. The biological fluid entering the polymer occupies the empty space that exists between the macromolecules of the polymer. The structure of the polymer becomes more compact, while the mobility of the macromolecules is more limited. Consequently, more energy is required to transition from the rigid state to the elastomeric state. In this case, the T_g_ value increases.
  - The third phenomenon has to do with the chemical interaction of water present in the biological fluid with the polymer. Water creates hydrogen bonds with the macromolecules of the polymer. Consequently, the mobility of macromolecules is limited. Therefore, more energy is required to transition from the rigid state to the elastomeric state. When this happens, the T_g_ value increases.

The plot of the T_g_ vs. the time of immersion shown in Figure 3 is the result of these three main phenomena, which act simultaneously and competitively, accompanied by other processes, depending on the thermoplastic type. The saturation of the voids, which changes the density of the polymer, occurs much faster than the plasticization of the polymer matrix. Therefore, in both the static and the dynamic stirring experiments, an initial increase in T_g_ was observed. The leaching phenomenon is further considered, where events start from the interior of the material. The process of leaching/migration of additives from plastics can be categorized into four steps, as follows: (1) diffusion leads to the additive moving towards the surface of the polymer; (2) desorption occurs as the additive separates from the polymer surface; (3) sorption takes place as the additive becomes absorbed within the plastic matrix in the surrounding medium; and (4) dispersion and absorption occur within the matrix [29,30]. The immediate effect is a decrease in T_g_. After this decrease, as observed in Figure 3, the T_g_ values for both the static and the dynamic stirring samples are equal. This happens on days 17 and 18, which is just after the samples’ weight also reached a similar value (see Figure 2), indicating that there is a logical correlation between the structure/weight of the static and dynamic stirring samples and their T_g_.

Furthermore, in static immersion, the leaching layer prevents water from penetrating the polymer. Therefore, in static immersion, after some time, the T_g_ maintains an almost constant value. The opposite happens to the dynamic stirring samples. The leaching layer detaches faster and, at the same time, the biological fluid penetrates at a greater rate into the polymer, which leads to a decrease in T_g_. The rotational forces prevent and delay the attachment of biochemicals to the surface of the PLA layer lines, while also contributing to the elimination of the leaching layer from the surface of the sample.

To summarize and correlate events, we found the following:
  - At the point of the intersection of the curves (day 17), it may be deduced that, in static immersion, the layer has formed, the liquid can no longer penetrate the polymer—or rather the penetration rate becomes very small—and, from then on, the T_g_ value reaches a plateau and remains almost stable.
  - In stirred immersion, the layer detaches, and the liquid penetrates the polymer matrix. The factor of plasticization dominates other phenomena; in addition, since the voids become saturated and the hydrogen bonds are weak, the T_g_ value is reduced.

### 4.4. Dynamic Mechanical Analysis (DMA) Discussion

From the graphs (Figure 4), we can observe the difference in the mode of variation of E′, depending on the conditions of immersion. More precisely, in the case of static immersion (Figure 5a), an initial increase in E′, followed by a subsequent decrease with the time of immersion, is observed; while, in the case of stirring conditions (Figure 5b), the decrease in E′ follows a linear variation with the time of immersion. Moreover, after 10 days of immersion, both curves become parallel to each other. In addition, as shown in Figure 5b, an initial 10% improvement in E′ is observed in the case of static immersion, followed by a subsequent continuous linear degradation after 28 days of immersion, reaching its maximum value of −15%. On the contrary, in the case of specimens under stirring conditions, a linear degradation of E′ of a maximum value of −40% is observed. Both types of E′ variation are compatible with the mechanisms already described above.

### 4.5. Discussion on the DMA and DSC Comparison

We can observe that the T_g_ values derived from the two methods are very close to each other, and that the trend of variation of the T_g_ values with the immersion time is identical. The curves are parallel to each other, with a small difference in T_g_ values. These findings show that the PLA behavior is constant, while the mechanism already explained in detail above on which it is based is independent of the experimental method applied for the evaluation of the specimens’ T_g_ when under static or stirring conditions.

## 5. Conclusions

In the present work, 3D-printed PLA scaffolds have been investigated with respect to their behavior when exposed to the following two types of micro-environments: a static one, which is commonly applied for the investigation of the biodegradation rate, and a stirring one, which was applied in the present investigation to biomimick the dynamic environment of the human body. Both experiments were performed at human body temperature. Moreover, a cell culture medium that contains several physiological components (enzymes, proteins, antibiotics, etc.) has been used as an immersion fluid. The cumulation effect of the biomimetic conditions results in more accurate feedback of a scaffold when it is subjected to real physiological conditions. The following conclusions have been drawn:

- The analysis of the SEM images performed on the scaffolds after different immersion times showed that the scaffolds in the static experiment visibly and considerably degrade after 28 days of immersion, while the scaffolds in the dynamic experiment are subjected to layer line breakage.
- The speed of weight reduction in stirred immersion is greater than the corresponding one in static immersion.
- In both cases, Tg initially rises because the filling of the polymer voids with fluid happens more quickly than the other processes.
- The variation curves of the Tg vs. immersion time as derived from the DMA measurements for both fluid absorption conditions (Figure 6) are similar in shape to the respective curves as derived from the DSC experiments (Figure 3c).
- Finally, as illustrated in Figure 5b, the storage modulus showed a 10% improvement in the case of static immersion, followed by a constant linear degradation that peaked at −15% after 28 days of immersion. Conversely, a linear degradation of the storage modulus to −40% is noted for the specimens held under stirring conditions. Both types of E′ variation fit within the mechanisms described.

## Data Availability

Data are available upon request.

## References

[1] Varma M.V., Kandasubramanian B., Ibrahim S.M. (2020). 3D printed scaffolds for biomedical applications. Mater. Chem. Phys..

[2] Zhan L., Wang L., Deng J., Zheng Y., Ke Q., Yang X., Zhang X., Jia W., Huang C. (2023). Enhanced cellular infiltration of tissue-engineered scaffolds fabricated by PLLA nanogrooved microfibers. Nano Res..

[3] Haycock J.W., Haycock J.W. (2011). 3D Cell Culture: A review of current approaches and techniques. 3D Cell Culture Methods in Molecular Biology.

[4] Londono R., Badylak S.F. (2015). Biologic scaffolds for regenerative medicine: Mechanisms of in vivo remodeling. Ann. Biomed. Eng..

[5] Dąbrowska-Gralak M., Sadło J., Głuszewski W., Łyczko K., Przybytniak G., Lewandowska H. (2022). The combined effect of humidity and electron beam irradiation on collagen type I—Implications for collagen-based devices. Mater. Today Commun..

[6] Yiwen X. (2022). Tribological Properties of Micro/Nano-Textured Surfaces Under Physiological Conditions. Ph.D. Thesis.

[7] Rouabhia M., Park H., Meng S., Derbali H., Zhang Z. (2013). Electrical stimulation promotes wound healing by enhancing dermal fibroblast activity and promoting myofibroblast transdifferentiation. PLoS ONE.

[8] Fatemi M., Manduca A., Greenleaf J.F. (2003). Imaging elastic properties of biological tissues by low-frequency harmonic vibration. Proc. IEEE.

[9] Chevalier J. (2006). What future for zirconia as a biomaterial?. Biomaterials.

[10] Hukins D.W.L., Mahomed A., Kukureka S.N. (2008). Accelerated aging for testing polymeric biomaterials and medical devices. Med. Eng. Phys..

[11] Zhao L., Zhang Z., Song Y., Liu S., Qi Y., Wang X., Wang Q., Cui C. (2016). Mechanical properties and in vitro biodegradation of newly developed porous Zn scaffolds for biomedical applications. Mater. Des..

[12] Carfì Pavia F., La Carrubba V., Brucato V. (2009). Tuning of biodegradation rate of PLLA scaffolds via blending with PLA. Int. J. Mater. Form..

[13] LeBlon C.E., Pai R., Fodor C.R., Golding A.S., Coulter J.P., Jedlicka S.S. (2013). In vitro comparative biodegradation analysis of salt-leached porous polymer scaffolds. J. Appl. Polym. Sci..

[14] Agrawal C.M., McKinney J.S., Lanctot D., Athanasiou K.A. (2000). Effects of fluid flow on the in vitro degradation kinetics of biodegradable scaffolds for tissue engineering. Biomaterials.

[15] Feier A.M., Portan D., Manu D.R., Kostopoulos V., Kotrotsos A., Strnad G., Dobreanu M., Salcudean A., Bataga T. (2022). Primary MSCs for personalized medicine: Ethical challenges, isolation and biocompatibility evaluation of 3d electrospun and printed scaffolds. Biomedicines.

[16] Mano J.F., Koniarova D., Reis R.L. (2003). Thermal properties of thermoplastic starch/synthetic polymer blends with potential biomedical applicability. J. Mater. Sci. Mater. Med..

[17] AnkerMake Explore the 3D Printing Frontier—Ankermake Europe. https://www.ankermake.com/eu-en/blogs/maintenance-guides/how-to-tell-if-filament-is-wet.

[18] Leyva-Porras C., Cruz-Alcantar P., Espinosa-Solís V., Martínez-Guerra E., Piñón-Balderrama C.I., Compean Martínez I., Saavedra-Leos M.Z. (2020). Application of differential scanning calorimetry (DSC) and modulated differential scanning calorimetry (MDSC) in food and drug industries. Polymers.

[19] Sun W.Q. (2021). DSC Analysis of thermophysical properties for biomaterials and formulations. Methods Mol. Biol..

[20] Mano J.F., Reis R.L., Cunha A.M. (2000). Effects of moisture and degradation time over the mechanical dynamical performance of starch-based biomaterials. J. Appl. Polym. Sci..

[21] Jones D.S. (1999). Dynamic mechanical analysis of polymeric systems of pharmaceutical and biomedical significance. Int. J. Pharm..

[22] Portan D.V., Kroustalli A.A., Deligianni D.D., Papanicolaou G.C. (2012). On the biocompatibility between TiO_2_ nanotubes layer and human osteoblasts. J. Biomed. Mater. Res..

[23] Portan D.V., Deligianni D.D., Deligianni K., Tyllianakis M., Papanicolaou G.C. (2018). Modeling of the interaction between osteoblasts and biocompatible substrates as a function of adhesion strength. J. Biomed. Mater. Res..

[24] Portan D.V., Ntoulias C., Mantzouranis G., Fortis A.P., Deligianni D.D., Polyzos D., Kostopoulos V. (2021). Gradient 3D printed PLA scaffolds on biomedical titanium: Mechanical evaluation and biocompatibility. Polymers.

[25] Odelius K., Höglund A., Kumar S., Hakkarainen M., Ghosh A.K., Bhatnagar N., Albertsson A.C. (2011). Porosity and pore size regulate the degradation product profile of polylactide. Biomacromolecules.

[26] Lam C.X.F., Savalani M.M., Teoh S.H., Hutmacher D.W. (2008). Dynamics of in vitro polymer degradation of polycaprolactone-based scaffolds: Accelerated versus simulated physiological conditions. Biomed. Mater..

[27] Bengi Y., Pazarceviren A.E., Tezcaner A., Evis Z. (2020). Historical development of simulated body fluids used in biomedical applications: A review. Microchem. J..

[28] Domingos M., Chiellini F., Cometa S., De Giglio E., Grillo-Fernandes E., Bártolo P., Chiellini E. (2010). Evaluation of in vitro degradation of PCL scaffolds fabricated via BioExtrusion. Part 1: Influence of the degradation environment. Virtual Phys. Prototyp..

[29] Iftikhar A., Qaiser Z., Sarfraz W., Ejaz U., Aqeel M., Rizvi Z.F., Khalid N. (2024). Understanding the leaching of plastic additives and subsequent risks to ecosystems. Water Emerg. Contam. Nanoplast..

[30] Li Y., Liu C., Yang H., He W., Li B., Zhu X., Liu S., Jia S., Li R., Tang K.H.D. (2024). Leaching of chemicals from microplastics: A review of chemical types, leaching mechanisms and influencing factors. Sci. Total Environ..

